# Primidone Intolerance in Essential tremor: Is it More than Just Age?

**DOI:** 10.5334/tohm.672

**Published:** 2021-12-31

**Authors:** Abhishek Lenka, Elan D. Louis

**Affiliations:** 1 Department of Neurology, MedStar Georgetown University Hospital, Washington, DC, USA; 2 Department of Neurology, University of Texas Southwestern, Dallas, TX, USA

**Keywords:** Essential tremor, treatment, primidone, side effects, GABA

## Abstract

**Background:**

There are few medications for the treatment of essential tremor (ET). One of these, primidone, which is one of only two front-line agents, is associated with considerable adverse drug reactions (ADRs). It is unclear why some primidone-treated ET patients develop ADRs whereas others do not, and why these ADRs seem to be more prevalent in ET patients than primidone-treated patients with epilepsy.

**Objective:**

To review several possible explanations underlying the above-referenced differences.

**Methods:**

A literature search was conducted in PubMed in October 2021. Studies reporting the ADRs of primidone in different neurological conditions were comprehensively reviewed.

**Discussion:**

Although there were no head-to-head data, a review of the previous studies on ET and epilepsy patients indicates that the former is relatively more intolerant to primidone. Moreover, not all ET patients develop ADR of similar nature or severity. We discuss several potential mechanisms for this variability in the intolerance to primidone. These include: (i) older age (ET vs. epilepsy patients), (ii) cross-tolerance to primidone in patients with epilepsy, (iii) neurobiological (GABA-related) abnormalities associated with ET.

**Conclusion:**

We speculate that there are several possible explanations for primidone intolerance in ET. These possibilities should be tested in future studies, and we propose the roadmap for designing these studies. It is of value to obtain detailed insight into these complex issues because primidone remains one of the few frontline anti-tremor medications in ET. Answers to issues we have raised in this article could facilitate more customized formulation of primidone in ET patients.

## Introduction

Essential tremor (ET) is among the most commonly encountered movement disorders in clinical practice [1,2]. Significant progress in the ET field over the last several decades has substantially improved our understanding of its epidemiology and etiopathogenesis. However, treatment options for ET remain limited. Oral therapeutic options include only two frontline medications, which either act on the gamma amino butyric acid-ergic (GABA-ergic) system (e.g., primidone) or as beta blockers (e.g., propranolol) [3]. There are two major drawbacks associated with these medications: (i) tremor-suppression is not achieved in all patients, and (ii) they are associated with a number of bothersome adverse drug reactions (ADRs). Approximately one-third of the ET patients discontinue their anti-tremor medications, probably due to perceived suboptimal medication efficacy and/or the bothersome ADR [4].

Intolerance to the GABAergic medications, especially primidone, is common and it is primarily due to ADRs such as sedation, nausea, vomiting, dizziness, vertigo, and disequilibrium. Numerous studies over the past 40 years have documented the presence of these ADRs in ET patients treated with primidone (***Table 1***) [5,6,7,8,9,10,11,12,13,14,15]. Interestingly, one group of authors anecdotally noted that the ADRs reported in ET patients taking primidone were more prevalent, even with low dose of primidone, compared to those seen in their primidone-treated patients epilepsy [5]. It is unclear why some primidone-treated ET patients develop ADRs whereas others do not, and why these ADRs are more prevalent in ET patients compared to primidone-treated epilepsy patients. The older age of ET patients relative to patients with epilepsy is presumed to be one of the contributing factors [16], although this remains somewhat speculative. There may be other explanations as well. Yet, to our knowledge, these have never been reviewed in a manuscript focused on this topic.

**Table 1:** Summary of the studies that reported adverse effects of primidone and/or PEMA in patients with essential tremor.


AUTHORS AND PUBLICATION YEAR	STUDY TYPE	NUMBER OF PATIENTS	AGE (YEARS)	DOSE	NUMBER OF PATIENTS WITH ADR (%)	ADR DETAILS

O’Brien et.al.1981 [5]	OL	20	15–78	Not mentioned	6 (30)	Vertigo, unsteadiness, nausea (Details of acute and chronic ADRs were not provided)

Chakrabarti and Pearce 1981 [6]	OL	5	47–80	125 mg/d for 2–3d (max 750–1000 mg/d)	0	No ADRs

Calzetti et.al.1981 [7]	RDBPC (PEMA)	8	28–69	week 1: 400 mg/d week 2: 800 mg/d	0	No ADRs

Findley and Calzetti 1982 [8]	RDBPC cross-over	11	15–82 (max 250 mg TID)	62.5 mg/d	4 (36.3%)	Sedation, tiredness, nausea, giddiness (Details of acute and chronic ADRs were not provided)

Findley et.al 1985 [9]	RDBPC cross-over (Extension of previous study) [8]	22	16–82	62.5 mg/d (max 250 mg TID)	5 (22.7%) withdrew due to ADRs (FDE). 11 (50%) had less severe ADRs	Nausea, vomiting, ataxia, giddiness (both acute and chronic toxicities had similar spectrum of ADRs)

Koller and Royse 1986 [10]	OL	22 (P+) 10 (drug-naïve)	56.5 (average) 52.3 (average)	50 mg at bedtime (max 1000 mg/d)	3 (9.3%) had FDE. 9 (32.1%) had Chronic ADRs.	FDE: Ataxia, confusion. Chronic: vertigo, sedation.

Dietrichson and Espen 1987 [11]	SBPC (Primidone vs propranolol)	13	34–77	62.5 mg/d (max 250 mg TID)	6 (46%) had FDE. 13 (100%) had chronic ADRs.	FDE: Ataxia, vomiting, blurred vision, headache, dry mouth. Chronic: nausea, dizziness, sedation.

Seyfert et.al. 1988 [12]	OL	11	29–68	single dose 750 mg	11 (100% had FDE).	FDE: nausea, postural disturbance, headache, confusion. No chronic ADRs were Reported.

Sasso et.al. 1988 [13]	DBPC cross-over (primidone vs. phenobarbital)	16	60–78	62.5 mg (max 250 mg TID)	10 (62.5%) not specified how many had FDE and chronic ADRs	FDE: Nausea, vertigo. Chronic: sedation and fatigability.

Sasso et.al. 1990 [14]	OL	11	63–77	62.5 mg (max 250 mg TID)	8 (72.7%) had FDE. 72.7%, 36.3%, and 18.1% had chronic ADR at 3, 6, and 12-months, respectively.	FDE: malaise, headache, dizziness, drowsiness, nausea vomiting. Chronic: drowsiness, dry mouth, sexual dysfunction.

Nida et.al. 2015 [15]	OL (study on VT patients)	30	71.9±11.8	25 mg/d (increased by 25 or 50 mg/month as tolerated)	22 (73%) not specified how many had FDE and chronic ADRs. 9 (31%) stopped treatment due to ADRs.	Fatigue, nausea, headache, dizziness (details of FDE or chronic toxicity were not specified)


**ADR:** Adverse drug reactions; **FDE:** First dose effect; **Max:** Maximum; **mg/d:** milligrams/day; **OL:** Open label; **P+:** Patients on propranolol; **PEMA:** Phenyl ethyl malonamide; **RDBPC:** Randomized double blinded placebo-controlled trial; **SBPC:** Single blinded placebo-controlled trial; **TID:** Three times/day **VT:** Voice tremor.

As primidone is one of few effective oral medications for ET, it would seem worthwhile to revisit these issues. More specifically, is primidone intolerance in ET merely a function of age, other factors, or is it a function of something about the underlying disease itself? Do available published data help us answer this question?

## Method of Literature Search

We conducted a literature search in PubMed in October 2021 using several keywords and combinations. The keywords “essential tremor”, “epilepsy”, and “seizure” were combined with “primidone”. We excluded studies if they were not on humans, or were not published in the English language, or if the full texts were unavailable. We reviewed in detail the studies that provided data on side effects and tolerability of primidone. We assessed reference sections of these articles for additional studies pertinent to this review topic and also obtained articles from the authors’ personal collections. ***Table 2*** summarizes the details of the literature search.

**Table 2:** Results of search for articles from PubMed using various key words and their combinations.


KEY WORDS AND COMBINATIONS	NUMBER OF PUBLICATIONS		

	TOTAL IDENTIFIED	INCLUDED	EXCLUDED

“Essential tremor” AND “Primidone”	168	22	146 (NE+NH = 37, unrelated to the review = 109)

“Epilepsy” AND “Primidone”	854	16	838 (NE+NH = 289, unrelated to the review = 549)

“Seizure” AND “Primidone”	185	2	183 (NE+NH = 57, unrelated to the review = 126)

*Total number of articles included for review after removing the duplicates*			*33*

*Total number of articles included from the reference sections of the shortlisted articles*			*8*

*Total number of articles obtained from the authors’ personal collection*			*11*

***Final total number of articles included for review***			***52***


NE: Non-English language, NH: Article on non-human subjects.

## Basic Pharmacology of Primidone

Primidone is classified as a barbiturate, and more specifically, a deoxybarbiturate. Its two metabolites are phenobarbital and phenyl-ethyl-malonamide (PEMA) [17]. Primidone itself has a little effect on GABA receptor; however, phenobarbital has GABA-ergic properties. Phenobarbital exerts its GABA-ergic effect by increasing the duration of opening of the chloride channels in the GABA-A receptors, resulting in membrane hyperpolarization. Because of this mechanism, primidone increases the seizure threshold and hence, it has been used as an anti-seizure medication. Primidone was also repurposed for the treatment of tremor in the 1980s and since then, it has remained one of the frontline anti-tremor medications. Although the exact anti-tremor mechanism of action remains elusive, previous studies have suggested that the anti-tremor effect is probably due to the parent drug itself [13] and one of its metabolites i.e., phenobarbital [14] whereas the other metabolite, PEMA, does not have an anti-tremor effect [8]. The half-life of primidone is 5–10 hours whereas the half-lives of PEMA (1–2 days) and phenobarbital (4–5 days) are substantially longer. Some reports suggest that phenobarbital, in high doses, through unknown mechanisms, can be metabolized back to its prodrug primidone [18]. The anti-tremor effect of primidone is usually apparent within one hour of oral administration and it peaks between 2 and 7 hours after administration. Primidone suppresses limb tremor more effectively than cranial tremors (i.e., head tremor and voice tremor) [10], but the overall tremor suppressing effect is on par [19] or more than that with propranolol [12]. It has been observed that primidone at a dose of 250 mg/d results in tremor-suppression as much as with doses higher than 250 mg/d [11,20]. The tremor-suppressing effect is not universal and may not always correlate with the serum levels of the parent drug or its metabolite [21,22]. It has been reported that the anti-tremor effect of primidone may wean over time in some ET patients [11,23].

## Adverse Effects of Primidone

### Adverse Effects in Patients with ET

Several open-label and controlled studies of primidone in ET have documented improvement in tremor using both subjective and objective assessments of tremor. However, those studies have invariably documented the emergence of adverse effects in many ET patients on primidone (***Table 1***). While sedation is a common ADR, and it is usually dose-related, some patients develop other neurotoxic effects such as nausea, vomiting, dizziness, and ataxia [21]. In addition, rare occurrence of connective tissue disorders and a lupus-like illness, as a side effect of long-term use of primidone, have been reported [24,25]. Almost all studies have reported primidone-related ADRs and, based on the timing of onset, these ADRs may be divided into two types. First, many patients develop ADRs (predominantly sedation and nystagmus) during the first day of administration of primidone (“first-dose effect”) and this is often but not always seen in the context of a high initial dose [26]. In fact, primidone with a dose as low as 2.5 mg three times daily also resulted in bothersome ADRs in ET patients in a study [27]. Seven of 20 patients who were on such a low dose experienced some sort of side effects within 48-hours and four patients discontinued primidone before 21-days [27]. Second, patients may develop chronic ADRs with continued treatment with primidone. The first-dose effects usually subside within a few days as patients develop tolerance to the medication [28]. One of the previous studies had concluded that the initial ADRs subsided presumably due to functional tolerance (decrease in sensitivity of the central nervous system to the drug) rather than adaptive tolerance (adaptive change in drug disposition) [28]. Unfortunately, most studies have not explicitly documented the pattern, severity, and time course of the first-dose effect associated with primidone. Since its introduction as an anti-tremor medication in 1981, only one study reported ADR-free improvement in all treated ET patients [6]. All of the remaining studies have documented ADRs with the use of primidone, albeit with considerable variability.

### Adverse Effects in Patients with Epilepsy and Other Disorders

Similar types of primidone-related ADRs have been reported in patients with epilepsy, as described below. A study of 392 pediatric patients assessed ADRs associated with several anti-seizure medications, including primidone, carbamazepine, phenytoin, valproate, and phenobarbital [29]. Interestingly, contrary to the observation of considerable ADRs in ET patients (***Table 1***), the frequency of ADRs was the lowest in patients on primidone monotherapy (29%) when compared with the other medications mentioned above. Dose adjustment because of ADRs was necessary in only 8% of the primidone treated epilepsy patients. In an earlier study that compared the efficacy and ADRs of primidone on epileptic and non-epileptic patients with psychiatric symptoms, doses up to 1,500 mg were administered [30]. While the authors had documented some ADRs only as a “first-dose effect”, those symptoms (dizziness, drowsiness, nausea) resolved within few days and there were not bothersome chronic effects. Interestingly, primidone was discontinued only in one patient, and that was because of an uncommon ADR i.e., morbilliform rash. The three patients who were reported to have the classic ADRs of primidone (drowsiness, dizziness, nausea) were aged 16, 24, and 31 years (average 23.6 years). On the contrary six patients did not experience any ADRs and they were relatively older (14, 37, 40, 41, 43, and 48 years; average 37.2 years) [30]. In another study of 21 patients with non-idiopathic epilepsy, four (19.1%) patients (data on age not available) reported drowsiness and ataxia early during the course of primidone treatment [31]. However, it needs to be emphasized that dose of primidone was much higher (1,500 mg to 2,000 mg) in this study compared the conventional dose prescribed to the ET patients (maximum of 750 mg/d). In another study from 1950s, six children with epilepsy refractory to phenytoin received primidone, and primidone was gradually titrated up to a dose of 5.25 g/week [32]. The authors did not observe any adverse effects related to primidone. In a study on 38 patients with intellectual impairment and epilepsy (ages from 7–52 years), primidone doses were in a range of 1–2 g/d, and patients were reportedly free of any long-term adverse effects. Some patients had drowsiness and ataxia, which were brief-lasting and not bothersome [33]. In fact, given the lack of serious ADRs, the authors mentioned “*That primidone is relatively free from toxic effects is again demonstrated, and it may therefore be safely used in domiciliary practice*.” [33] In a study on 486 patients with epilepsy, ADRs were noted in 211 patients (43.4%) who received primidone, with a dose range of 1,000 mg to 2,000 mg/d [34]. However, the ADRs led to discontinuation of the drug only in 46 (9.4%) patients. The most common ADR was drowsiness (n = 160), followed by dizziness, ataxia, and nausea. A study in 1956 investigated the efficacy of primidone alone or in combination with chlorpromazine in patients with epilepsy and intellectual disability [35]. Of the 25 patients on primidone, only four (16.0%) had the typical ADR of primidone (disequilibrium, lethargy, slurred speech). However, the authors noted that patients who were on primidone had a subjective feeling of greater well-being, alertness, and optimism [35]. This is counterintuitive in the context of the well-known bothersome ADR of primidone i.e., drowsiness, dizziness, etc. Another study compared the efficacy of primidone and carbamazepine in 45 patients with epilepsy [36]. In that study the most common ADRs of primidone were drowsiness, unsteadiness, and nystagmus. However, none of these effects were permanent.

Data on ADRs associated with the use of primidone in diseases other than ET and epilepsy are limited. One study examined the efficacy of primidone for the treatment of tremor in multiple sclerosis [37]. While it effectively ameliorated tremor in the ten patients (age range 14–46 years), the ADRs were benign (only drowsiness) and none of the patients discontinued treatment because of adverse effects.

## Differences in the Tolerance to Primidone: Possible Causes

The variability in the pattern and severity of ADR as described above raises questions about its cause, and below we raise several possibilities.

As described above, epilepsy patients seem to exhibit a relatively lower burden of primidone-related ADRs when compared to ET patients, even though the former receive primidone at far higher dosages (up to 2g/d). It is described in the epilepsy literature that sedation is uncommon with primidone concentration lower than 10µg/ml (corresponding to 10 mg/kg/d) and ataxia or gait unsteadiness is uncommon with a concentration lower than 20 µg/ml (corresponding to 10 mg/kg/d) [22]. However, such doses (10 mg/kg or 20 mg/kg) are very high in the context of the treatment of tremor [20]. One possibility is that this difference is *age-related*. ET patients in the primidone treatment studies were considerably older than the epilepsy patients, many of whom were children or young adults. It is possible that the pharmacodynamics and pharmacokinetics of primidone differ significantly in younger and older patients, presumably due to age-related changes in blood-brain permeability, total body water, lean body mass, and kidney and liver function [38]. It is also possible that tolerance is related to age-related changes in the brain and/or cognitive reserve. Assessing whether age is an important factor in the observed difference between ET and epilepsy patients is a challenge, however. While there is an abundance of published data on ADRs in older ET patients and younger epilepsy patients, published data on ADRs in young ET patients and older epilepsy patients are limited. A second possibility relates to *cross-tolerance*. A few studies speculated that prior or concurrent use of other enzyme-inducing anti-epileptic medications might result in a relative cross-tolerance to primidone, thus, resulting in fewer ADR, including the first-dose effect, in epilepsy patients [39,40]. Phenobarbital pre-loading is sometimes used in ET patients to improve their tolerance to initiation of primidone therapy. However, we do not have robust data to unequivocally support the cross-tolerance theory. While sedation has been the most commonly reported ADR of primidone in humans, counterintuitively, a study on rats revealed that injection of primidone provoked wakefulness and reduced the duration of slow wave sleep and rapid eye movement sleep [41]. While additional studies need to confirm this unexpected effect of primidone in rats, the result of this study certainly adds to the controversies related to the pharmacological properties of primidone. Interestingly, similar findings were documented in a study on children with epilepsy [35].

There is a third possibility. As noted above, it is possible that the intolerance to primidone in ET patients is directly related to the *neurobiological abnormalities associated with ET*. This discussion can take two forms. First, there is accumulating evidence that ET patients may also develop a wide-range of non-motor symptoms (NMS) [42,43]. Sleep disturbance is a common NMS of ET and both insomnia as well as excessive daytime sleepiness (EDS) have been reported in previous studies [42]. Moreover, ET patients with cognitive impairment may have more EDS compared to those with preserved cognition [44]. Fatigue is also a commonly reported NMS in ET and it negatively affects the quality of life of ET patients [45,46]. Interestingly, these two NMS of ET i.e., EDS and fatigue, have also been reported as two common ADRs of primidone. Therefore, it may be speculated that ET patients with EDS and fatigue might be more susceptible to the ADR of primidone due to a cumulative effect of the “disease” (ET) and “drug effect” (primidone). There are no data at present, however, to support this speculation. ET patients may also develop other non-tremor motor features, and one of these is ataxia. Since disequilibrium is also a commonly reported ADR of primidone, it may be speculated that ET patients with mild ataxia, when exposed to primidone, may develop sudden worsening of the ataxia. It needs to be emphasized that such gait abnormalities are not uncommon in older ET patients and their progression may be related to poor cognition [47,48]. However, there are no data at present to support this speculation. In summary, certain features occur in ET, including cognitive impairment, EDS, fatigue, and ataxia, and the patients who suffer from these, could be at higher risk for ADR; again, data need to be generated to support these hypotheses.

As noted above, we raised the possibility above that the intolerance to primidone in ET patients is directly related to the *neurobiological abnormalities associated with ET*. We noted that this discussion could take two forms. One alternative possibility is there is considerable evidence that there is an abnormality in GABA neurotransmission in ET, and this may relate to the pathology observed in the ET cerebellum [49,50,51,52]. Given a disordered GABA substrate in ET, it may be that a medication that interfaces with the GABA system (i.e., primidone) is not tolerated as well in this disease.

Along with these possible explanations raised above, one other factor should be considered and further researched. Both ET and epilepsy are chronic diseases often requiring longstanding treatment. However, untreated epilepsy is generally associated with greater morbidity and mortality compared to untreated ET. As such, the epilepsy population may be more accepting and tolerant of ADR’s than the ET population, leading to a possible relative underreporting of ADRs in the former.

## Conclusions and Roadmap for Future Studies

**Figure 1:**
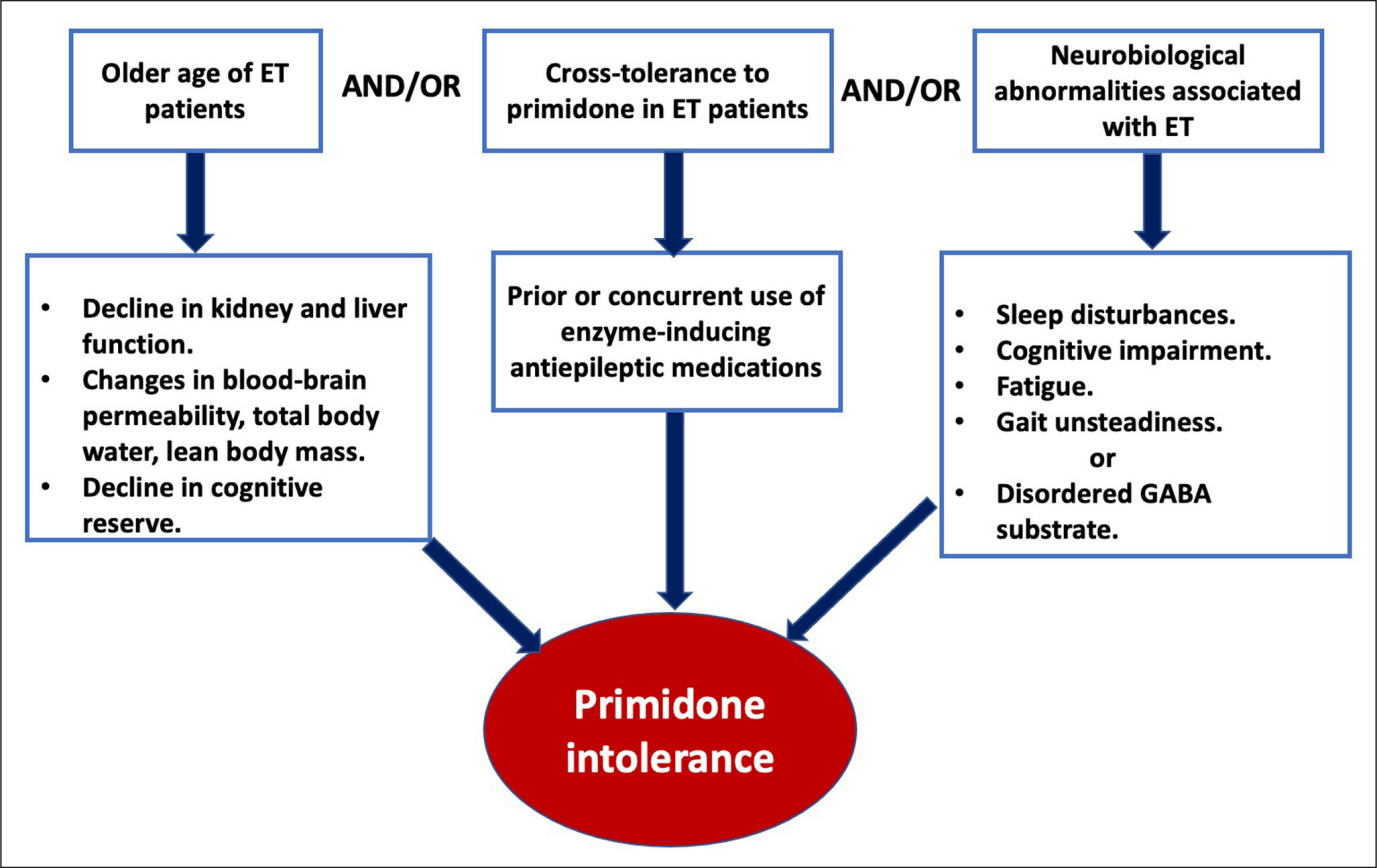
Summary of the factors that could potentially explain the primidone intolerance in essential tremor relative to patients with epilepsy.

Although the literature on primidone intolerance in ET is limited, careful review of the available literature reveals that the frequency, spectrum, and severity of the ADRs associated with primidone use in ET are highly variable. Moreover, ADRs associated with primidone use in ET are more frequent and more severe compared to those in patients with epilepsy. We speculate that there are several possible reasons that can explain the primidone intolerance in ET relative to patients with epilepsy. Those include (i) the older age of ET patients, (ii) cross-tolerance to primidone in patients with epilepsy, (iii) neurobiological abnormalities associated with ET. (***Figure 1***). These hypotheses need to be tested in future studies and we propose the roadmap for the same (***Table 3***). It is of value to obtain detailed insight into this complex issue because effective treatment of ET has remained an unmet need and primidone still remains one of the few frontline anti-tremor medications in ET. Answers to issues we have raised in this article could facilitate more customized formulation of primidone in ET patients.

**Table 3:** Strategies for future studies to explore the neurobiology of primidone intolerance in ET.


• Prospective study exploring the emergence of primidone-associated ADRs in ET patients with and without certain clinical features including fatigue, excessive daytime sleepiness, ataxia, and cognitive impairment.

• Comparison of ADR profile of primidone in younger and older ET patients.

• Structural and functional neuroimaging studies on ET patients with and without primidone intolerance to explore neural correlates of such ADR.

• Large clinical trials to test the cross-tolerance theory (longitudinal assessment to explore for ADR after primidone treatment with and without pre-treatment with phenobarbital).

